# Genotypic and Phenotypic Characterization of Replication-Competent HIV-2 Isolated from Controllers and Progressors

**DOI:** 10.3390/v15112236

**Published:** 2023-11-09

**Authors:** Cynthia Lungu, Ronald J. Overmars, Esmée Grundeken, Patrick H. M. Boers, Marchina E. van der Ende, Thibault Mesplède, Rob A. Gruters

**Affiliations:** 1 Viroscience Department, Erasmus Medical Center, Wytemaweg 80, 3015 CN Rotterdam, The Netherlands; c.lungu@erasmusmc.nl (C.L.); r.overmars@erasmusmc.nl (R.J.O.); grundeken.e@gmail.com (E.G.); patgoesitaly@hotmail.com (P.H.M.B.); 2 Department of Internal Medicine, Erasmus Medical Center, Wytemaweg 80, 3015 CN Rotterdam, The Netherlands; inekevanderende@xs4all.nl

**Keywords:** HIV-2, long-term non-progressors, transactivation, Tat

## Abstract

Although some individuals with HIV-2 develop severe immunodeficiency and AIDS-related complications, most may never progress to AIDS. Replication-competent HIV-2 isolated from asymptomatic long-term non-progressors (controllers) have lower replication rates than viruses from individuals who progress to AIDS (progressors). To investigate potential retroviral factors that correlate with disease progression in HIV-2, we sequenced the near full-length genomes of replication-competent viruses previously outgrown from controllers and progressors and used phylogeny to seek genotypic correlates of disease progression. We validated the integrity of all open reading frames and used cell-based assays to study the retroviral transcriptional activity of the long terminal repeats (LTRs) and Tat proteins of HIV-2 from controllers and progressors. Overall, we did not identify genotypic defects that may contribute to HIV-2 non-progression. Tat-induced, LTR-mediated transcription was comparable between viruses from controllers and progressors. Our results were obtained from a small number of participants and should be interpreted accordingly. Overall, they suggest that progression may be determined before or during integration of HIV-2.

## 1. Introduction

Untreated HIV-1 infection typically leads to the progressive depletion of CD4+ T-lymphocytes, resulting in weakened immune function, increased susceptibility to opportunistic infections, and death [1]. Less than 1% replication-competent integrated HIV-1 proviral copies within cellular reservoirs persists despite potent antiretroviral therapy and fuels viral rebound after treatment interruption [2,3]. Individuals who control HIV-1 infection without treatment are rare [4]. They are typically split into different groups, including aviremic elite controllers (ECs) who have no or little detectable viral RNA in their plasma and no CD4+ loss, viremic long-term non-progressors (LTNPs) with detectable viral loads but no CD4+ loss, and post-treatment controllers [5,6]. The precise mechanisms underlying the control of HIV-1 remain unclear [6]. However, host factors, specific CD4+ and CD8+ T cell immune responses, and specific HLA class I alleles are known to play significant roles [7,8,9]. Additionally, intrinsic viral characteristics can also contribute to virologic control, including deletion in the long-terminal repeat and Nef [10,11], envelope sequences [12,13], low viral fitness [14], and the nature of integration sites [10,11,12,15] (reviewed in [6]). Gaining a deeper understanding of the mechanisms governing HIV control could potentially provide valuable insights into interventions aimed at enabling viral control in all people with HIV without the need for antiretroviral therapy.

HIV-2 is markedly less virulent than HIV-1, with an extended asymptomatic phase that can span over 20 years and a decreased likelihood of progressing to AIDS [16,17]. Notably, while some persons with HIV-2 develop severe immunodeficiency and AIDS-related complications, a majority experience a better prognosis and may never progress to AIDS in their lifetime, thus qualifying as ECs or LTNPs [18,19,20,21]. In contrast, HIV-1 ECs are rare, constituting only ~1% of cases [22]. Given the rarity of this phenotype in HIV-1 infection, studying HIV-2 non-progression may be more feasible [20,21,23,24]. It is important to emphasize that this relative attenuation of HIV-2 does not mean that HIV-2 infection is not deleterious to the host; rather, HIV-2 infection is associated with disease and death [25,26].

One of the main differences between HIV-1 and -2 infection is that the accumulation of viral mRNA is lower in HIV-2-infected cells relative to HIV-1-infected cells [27,28]. Some of this difference can be recapitulated in cell culture experiments using monocyte-derived macrophages, where HIV-1 and HIV-2 exhibit different replication kinetics [28]. Given that plasma viral loads correlate positively with disease progression, HIV-2 replication may be restricted in vivo compared to that of HIV-1 [21,27]. In addition, total cellular and integrated proviral DNA levels are similar in HIV-1- and HIV-2-infected individuals matched for disease stage [27,29]. Hence, it is conceivable that the HIV-2 provirus is subjected to more stringent transcriptional control than HIV-1 [27]. In agreement with this hypothesis, cell-based experiments recently showed that HIV-2 replication is less cytotoxic but also less reactivable than HIV-1 [30]. Alternatively, HIV-2 might be more susceptible to immune pressure in vivo, resulting in better elimination of HIV-2 productively infected cells. 

Importantly, we previously reported that replication-competent HIV-2 isolated from asymptomatic HIV-2 controllers exhibited lower replication rates than viruses from individuals with HIV-2 who progressed to AIDS, indicating an association between in vitro replication capacity and disease progression akin to that observed for HIV-1 [31,32,33,34,35]. Using GHOST indicator cells, we demonstrated that HIV-2 employs a diverse range of co-receptors for entry, including CCR5, GPR15 (BOB), and CXCR (BONZO), with CXCR4 utilization observed only in HIV-2 variants from progressors [36]. We also showed that Nef proteins from HIV-2 controllers and progressors efficiently downmodulate TCR-CD3, CD28, and MHC-1 from the cell surface, regardless of the individual’s phenotype [37]. Additionally, we established that both effective Vpx-mediated SAMHD1 degradation and enhancement of myeloid cell infection were similar across viruses from controllers and progressors [38]. The precise factors underpinning HIV-2 control thus remain incompletely identified.

In this study, we sequenced the near full-length genome of replication-competent HIV-2 isolated from a few controllers and progressors to pinpoint potential additional retroviral factors associated with HIV-2 control. Given the fact that replication-competent viruses isolated from progressors displayed higher replication rates than viruses from controllers, we reasoned that investigating the sequences of these viruses might reveal new insights relating to the in vivo phenotype. Sequences were made available to the research community on GenBank (Accession numbers MF595854-MF595866). Since the process of transcriptional regulation has been shown to contribute to viral control [27], we evaluated the genetic and phenotypic variability of the long terminal repeats (LTRs) and Tat in HIV-2 and their possible association with the replicative capacities of these retroviruses. Our research showed no genetic clustering of near full-length genomes or specific genes based on progression or control. Furthermore, genotypic functional defects such as premature stop codons were absent in all open reading frames (ORFs), including Env and Rev in which functional domains were conserved. In addition, functional assays demonstrated that Tat and LTR transcriptional activities were comparable for viruses isolated from controllers and progressors. Despite the small number of sequences, based on these results, we hypothesize that the reduced replicative capacity of HIV-2 from controllers compared to viruses from progressors might be ascribed to qualitative disparities in the integration phase of infection. Our future work will investigate this possibility. 

## 2. Materials and Methods

### 2.1. Participants’ Characteristics and HIV-2 Isolates

The study was approved by the Erasmus MC *Medisch Ethische Toetsings Commissie* (MEC-2000-221). All participants provided written informed consent. The persons referred to in this study (Table 1) are from a cohort of people with HIV-2 and either aviremic controllers with undetectable plasma viremia (n = 2) or progressors with detectable plasma viremia and progressive disease (n = 4), all attending the Rotterdam outpatient clinic, as described previously [39]. Viral loads and CD4+ T-cell counts are reported. All participants had a confirmed HIV-2 group A infection. The isolation of HIV-2 replication-competent viruses by co-cultivation of peripheral blood mononuclear cells (PBMCs) with HIV-negative donor CD8-depleted PBMCs in limiting dilution series was published previously [31]. The replicative capacities of the viruses used in this study were also previously tested [33]. All viral stocks described in this manuscript are available via the European Vaccine Archive program under Human Immunodeficiency Virus type II, RH-2-X with specific clone names as mentioned in Table 1 (https://www.european-virus-archive.com/, accessed on 1 November 2023).

### 2.2. DNA Isolation, Amplification, and Sequencing, and Sequence Analysis

Genomic DNA was isolated from infected cell (CD8-depleted PBMCs) pellets using a GenElute Mammalian Genomic DNA Miniprep kit (Sigma-Aldrich, St. Louis, MO, USA). Pfu Ultra II Fusion HS DNA polymerase (Agilent Technologies, Santa Clara, CA, USA) was used for PCR amplification of 3 overlapping regions (R1–R3) spanning the near full HIV-2 genome. Primers were designed to match conserved regions in HIV-2 group A and numbered according to nucleotide positions in the prototype virus, HIV-2 BEN. Region R1 was amplified using forward primer P1f (5′-TGGAAGGGATGTTTTACAGTGAG-3′), and reverse primer P3513r (5′-TGGAAGGCTARACTGAAAGCAAG-3′); R2 was amplified using forward primer P3485f (5′-TGGATGATATCTTAATAGCTAGTGACAGG-3′) and reverse primer P6420r (5′-CCTTCAAGGGTGTCTCCATGTC-3′); and R3 was amplified using forward primer P6402f (5′-ATGGAGACACCCTTGAA-3′) and reverse primer P9559r (5′-GTTACAGCCCCTTCTGGAAAGTC-3′). Each primer was used at a concentration of 0.2 μM in a 50 μL reaction volume. PCR products were purified from agarose gel using the QiaEx II Gel extraction kit (Qiagen, Hilden, Germany).

Purified PCR products were sequenced using BigDye Terminator (Applied Biosystems, Waltham, MA, USA) on an ABI Genetic Analyzer 3130 (Applied Biosystems). Contiguous sequences were assembled and visually inspected for quality using SeqMan Pro in DNASTAR software 10.1. Manual sequence editing and alignment by Clustal W was implemented in MEGA 7.0 [26]. All sequences were submitted to GenBank and are available under accession numbers MF595854.1 to MF595866.1.

### 2.3. Sequence Analyses

Near full-length sequences were checked for hypermutation with the Hypermut analysis tool from the Los Alamos National Laboratories (https://www.hiv.lanl.gov/content/sequence/HYPERMUT/hypermut.html, accessed on 23 February 2023). Maximum-likelihood phylogenetic trees were constructed using PhyML 3.0 (http://www.atgc-montpellier.fr/phyml, accessed on 17 July 2023). Reference sequences used in the alignments were obtained from the Los Alamos National Laboratory HIV database (https://www.hiv.lanl.gov, accessed on 17 July 2023). Phylogenetic trees were inferred based on a GTR+G+I model and verified by 1000 bootstraps [40]. Resulting trees were viewed and annotated in FigTree v1.4.3 (http://tree.bio.ed.ac.uk/software/figtree, accessed on 17 July 2023). Sequence pairwise distances were estimated using Clustal method in MEGA 7.0. Single-nucleotide polymorphisms were identified using Geneious Prime v. 2023.1.2 (Biomatters Ltd., Auckland, New Zealand).

Open reading frames were translated using MEGA 7.0. Translated sequences were annotated using data available at Uniprot (http://www.uniprot.org, accessed on 23 July 2023). N-glycosylation sites within the Env sequences were predicted using the N-glycosite tool available at the Los Alamos HIV database site (https://www.hiv.lanl.gov, accessed on 28 August 2023).

### 2.4. Plasmids

HIV-2 5′-LTR fragments (U3 to R region) were amplified from the above-described isolates using the SacIHIV2U3 (5′-ACTGGCCGGTACCTGAGCT*C*TGGAAGGGATGTTTTACAGTG-3′; SacI site and plasmid vector sequence are underlined) forward and XhoIHIV2R (5′-TCTTGATATCCTCGAGAAGCAAGCAAGCGTGGAG-3′; XhoI site and plasmid vector sequence are underlined) reverse primers. Purified PCR products were inserted into the SacI (+26) and XhoI (+34) restriction sites of the pGL4.10 [luc2] vector using an In-Fusion^®^ HD Cloning kit (Takara Bio, San Jose, CA, USA) according to the manufacturer’s instruction. The pGL4.10 produces firefly luciferase following transcriptional activation. With this approach, we successfully created the following plasmids (collectively named pGL4.10-LTRs): pGL4.10-LTR (RH2.3), pGL4.10-LTR (RH2.14) from controllers; and pGL4.10-LTR (RH2.5), pGL4.10-LTR (RH2.7), pGL4.10-LTR (RH2.21), and pGL4.10-LTR (RH2.24) from progressors. The pROD10 reference plasmid was used as a positive control, permitting the creation of the pGL4.10-LTR (ROD). Plasmids were verified by restriction enzyme digestion and sequencing. 

The plasmid pROD214 ([41], a gift from Ben Berkhout) encoding Tat was used for the initial transactivation assays described below. For experiments in which transactivation was measured with paired LTRs and Tat, Tat coding sequences from controllers and progressors were cloned into pROD214, in replacement of Tat_ROD_. Briefly, the full Tat coding region from RH2.3 and RH2.14 (controllers); and RH2.21 and RH2.24 (progressors) were successfully amplified using the 5′-GAAGAAGCTTTAARGCATTTTG-3′ 5778_5799_Tat forward and 5′-GCKTCTTGGATCCACTCG-3′ 8581_8564_Tat reverse primers. These primers contain a HindIII and BamHI restriction site (underlined), respectively, which we used to clone the amplicons into pROD214 following the removal of Tat_ROD_ using the same restriction enzymes. The resulting p214-Tat plasmids were checked by sequencing with the same primers and used in subsequent transactivation assays. 

### 2.5. Transactivation Assays

Initially, the transactivation assays were performed by co-transfecting discrete amounts of the pGL4.10-LTRs and pROD214 plasmids. In this assay setup, the transactivation of LTR-driven transcription was thus mediated by Tat_ROD_ for all LTRs. All transfections were carried out using polyethylenimine (PEI). Specifically, 30,000 293T cells per well were co-transfected on a white, 96 well, flat bottom, tissue culture plate (Costar, Washington, DC, USA) with 10 ng of pGL4.10-LTR; 2.5 ng of a Renilla luciferase reporter plasmid to normalize transfection efficiency; and 0, 5, 10, or 15 ng of pROD214. 293T cells were cultured in Dulbecco’s Modified Eagle Medium (DMEM) (Lonza, Bornem, Belgium) supplemented with 10% fetal calf serum (FCS), penicillin (100 U/mL), streptomycin (100 mg/mL), 0.01% non-essential amino acids (Lonza, Bornem, Belgium) and 0.01% Sodium pyruvate (Lonza, Bornem, Belgium) at 37 °C and 5% CO_2_. Luciferase activity was analyzed 48 h post-transfection using a Dual-Glo^®^ Luciferase Assay System (Promega, Madison, WI, USA) according to the manufacturer’s protocol. Bioluminescence was measured using an Infinite 200 luminometer (Tecan, Zurich, Switzerland). Relative basal LTR activity was arbitrarily set at a value of 1. Relative transactivation was calculated by dividing the relative Tat-induced LTR activity of each pGL4.10-LTR clone by its own relative basal activity. Experiments were repeated 3 times with experimental triplicates in each experiment.

Transactivation assays with matching Tat and LTRs were performed using a similar method, except that the 30,000 293T cells were co-transfected with pGL4.10-LTRs (10 ng) and p214-Tat plasmids (0, 7.5, 15, or 60 ng). Thus, transactivation of LTR transcription was mediated by the Tat from the same donor. The pGL4.10-LTR (ROD) and pROD214 combination was used as a control. Experiments with the matched LTR and Tat were performed 4 times with experimental triplicates. The experiments with the ROD control were performed 3 times in triplicates.

### 2.6. Statistical Analysis

Statistical analyses were performed using GraphPad v.7 (Prism Software, Irvine, CA, USA) and verified with OpenEPI (https://www.openepi.com/, accessed on 29 August 2023).

### 2.7. Molecular Modeling

Integrase sequences were used to generate in silico structures using ColabFold (AlphaFold2, v1.5.3) [42]. Top-ranked structures were aligned and scored using the pairwise structure alignment tool of the RCSB PDB (www.rcsb.org/alignment, accessed on 27 October 2023). HIV-2 Nef structure was obtained from the RCSB PDB (ID: 6K6M) [43]. Visualization, mutagenesis, and illustration were performed using the PyMol software version 2.3.2 (Schrodinger LLC, Mannheim, Germany), as previously published [44].

## 3. Results

### 3.1. Participants’ Characteristics

At the time of virus isolation (2001–2003), HIV-2 aviremic controllers (n = 2) had spontaneous virus suppression <50 RNA copies/mL of plasma for more than 15 years (Table 1). Participants RH2.3 and RH2.14 were first tested positive for HIV-2 in 1992 and 2000, respectively. In 2016, they remained with undetectable viral loads and no apparent decline in CD4+ T-cell counts. Participants who qualified as progressors (n = 4) had detectable plasma viral loads and low CD4 counts. At the time of virus isolation (2001–2003), only patient RH2.5 was receiving antiretroviral therapy. We have previously determined the in vitro replication rates of viruses isolated from all participants [33]. To further understand correlates of control, we performed near full-length sequencing of these viruses.

### 3.2. Genetic Association with HIV-2 Progression

**Figure 1:**
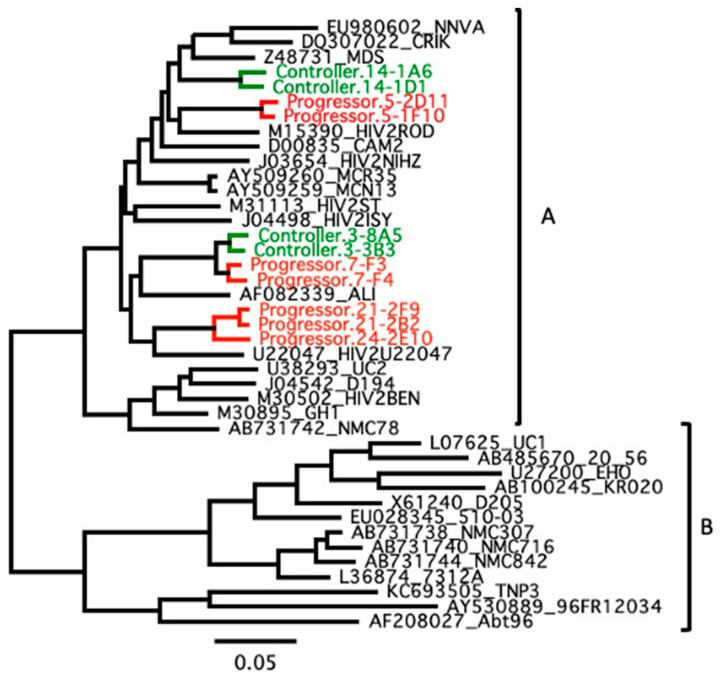
Maximum-likelihood phylogenetic tree of near full genome sequences of replication-competent HIV-2 isolated from donors and HIV-2 group reference sequences. HIV-2 sequences are shown in green when they were derived from controllers’ viruses and red from progressors. Sequences are labelled with “controller” or “progressor” followed by a patient ID number (“3”, “5”, “7”, “21” and “24”) and code identifying individual virus isolates (e.g., “1A6”). Genomes with identical patient ID number were derived from a single donor. Also included are HIV-2 group A and B reference sequences. Horizontal branch lengths are proportional to the scale bar at the bottom indicating nucleotide substitutions per site.

To investigate the genetic relatedness of the HIV-2 isolates in relation to biological phenotype, we reconstructed Maximum likelihood trees based on near full genome nucleotide sequences. As shown in Figure 1, viruses from controllers did not cluster together, e.g., viruses isolated from RH2.3 (controller) are closely phylogenetically related to viruses isolated from RH2.7 (progressor). We also reconstructed trees based on individual ORFs (gag, pol, env, vif, vpx, vpr, tat, rev, and nef) as well as the LTRs (not shown). These additional analyses yielded a similar distribution over the group A tree without clustering of the controllers’ sequences. As expected from replication-competent viruses, none of the sequences was found to be hypermutated and no premature stop codons were found in individual ORFs. Thus, no obvious haplotype contributed to HIV-2 control. 

**Figure 2:**
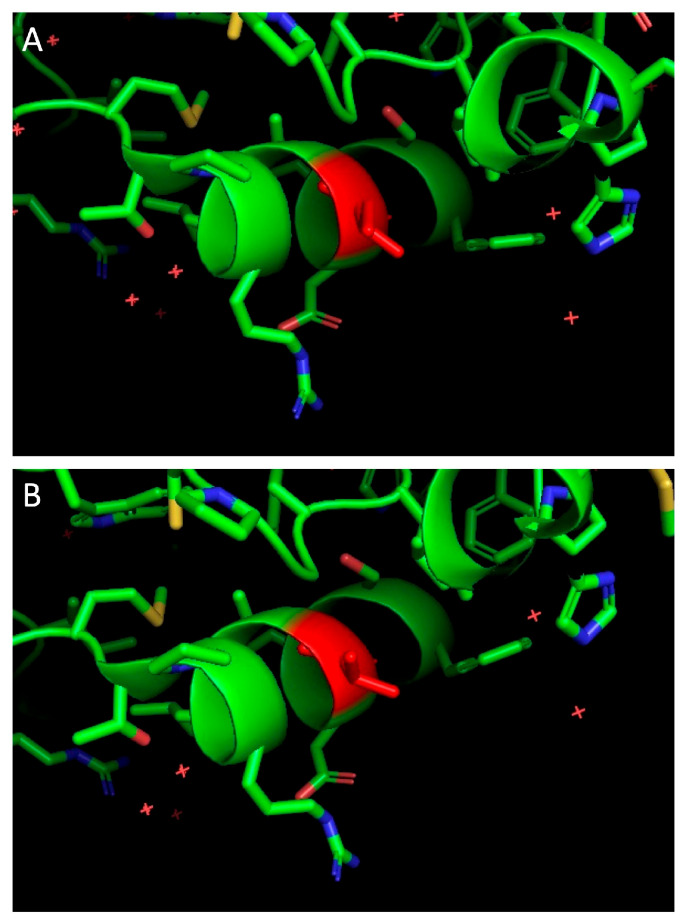
In silico structure modelling of HIV-2 Nef illustrating the residues I117 (**A**) and V117 (**B**). The overall structure of the protein remained unchanged. The residues of interest are shown in red.

However, individual single-nucleotide polymorphisms (SNPs) that do not contribute to the overall phylogeny of viruses may be functionally important. To further investigate this possibility, 1564 non-synonymous SNPs were identified and screened for their exclusive distribution within one or the other group of participants. Only two SNPs were found to be exclusively present in viruses from controllers, namely H413Q in Env and I117V in Nef. In silico structural modeling of I117 and V117 derived from the published HIV-2 Nef crystal structure (PDB ID:6K6M, [43]) did not show an obvious structural effect from this conservative substitution (Figure 2). The function of these two natural polymorphisms was not otherwise investigated.

### 3.3. Individual Proteins’ Contribution to Virologic Progression

**Figure 3:**
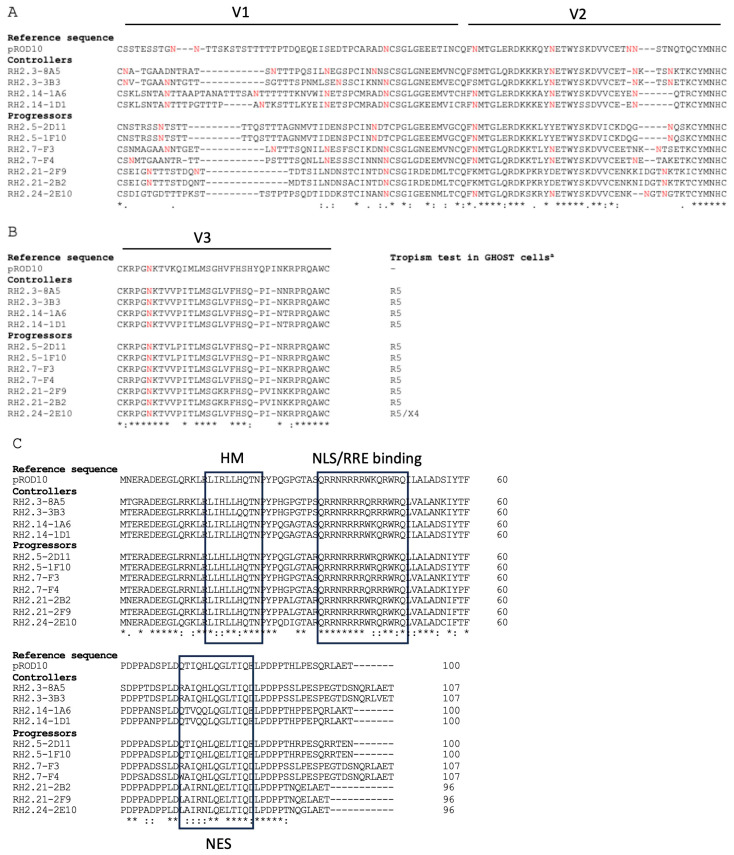
Conservation of functionally important residues and regions in Env and Rev of HIV-2 from controllers and progressors. (**A**) Amino acid sequence alignment of Env V1 and V2 hypervariable regions from controllers and progressors. Red letters indicate potential N-glycosylation sites based on the N-X-S/T motif, where X is any amino acid but P. (**B**) Amino acid sequence alignment of the Env V3 hypervariable region from controllers and progressors. The tropism indicated was previously published [36]. (**C**) Amino acid sequence alignment of Rev from controllers and progressors. The homo-multimerization region (HM), nuclear localization signal/Rev-response element-binding motif (NLS/RRE binding), and nuclear export signal (NES) are indicated. The HIV-2 ROD sequence (GenBank Accession ID:M15390) was used as a reference. (*) indicate amino acid conservation, (:) indicates conservative polymorphisms, (.) indicates semi-conservative polymorphisms, and (-) indicates a gap.

Next, we examined in detail the sequences of several retroviral proteins to compare them in progressors vs. controllers. We have previously functionally studied different Nef and Vpx proteins isolated from people with HIV-2 and showed that their functions did not correlate with virologic control [37,38]. Notable amino acid changes in Gag protein correlating with viral loads reported by Jallow and co-workers were also conserved [45]. The presence of this PPP-Gag motif has been linked to superior antigen processing and enhanced presentation to cytotoxic T lymphocytes (CTL). Strong gag-specific CTL-responses might thus explain the lower viral load for HIV-2 biological clones containing this motif in vivo [45], although it is unlikely to affect the cell-based replication rate in CD8-depleted PBMCs. Given the association between Env glycoprotein sequences and biological phenotype [46,47,48,49,50,51,52,53,54], we compared Env sequences from controllers and progressors (Figure 3). As shown in Figure 3A,B, no distinct patterns were observed in terms of the number of N-linked glycosylation sites within the V1-V2 and V3 regions of Env. Similarly, functional domains of Rev were conserved in all sequences (Figure 3C). 

It has been suggested that the presence of proline residues at position 119, 159, and 178 of capsid may contribute to low HIV-2 viral loads [55]. Thus, we also examined these residues but failed to find a clear association with control or progression (Table 2). The only viruses with three prolines at the three positions were isolated from a controller (RH2.3), but viruses isolated from the other controller (RH2.14) had the same amino acid distribution profile as viruses from a progressor (RH2.24).

**Figure 4:**
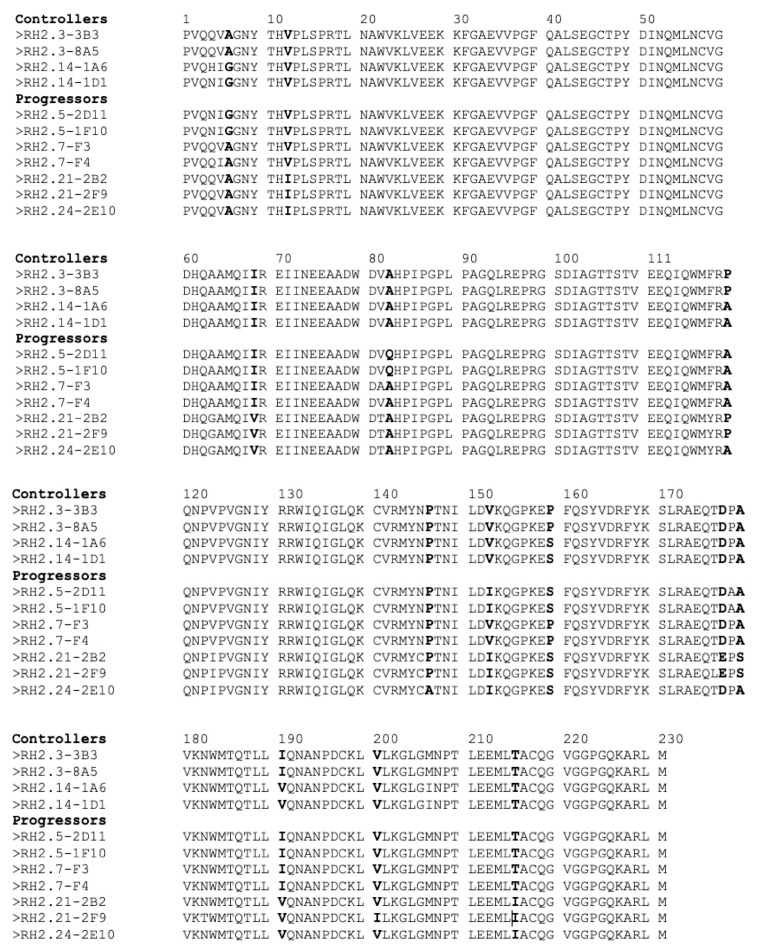
Conservation of residues in capsid of HIV-2 from controllers and progressors. Polymorphic residues are shown in bold, including positions 6, 12, 119, 159, and 178.

Another study identified capsid residues 6, 12, and 119 as important for fast progression [56]. In our small cohort, we did not observe a clear link between the nature of these 3 residues and progression (Figure 4).

### 3.4. Transcription Regulation in HIV-2 Controllers and Progressors

**Figure 5:**
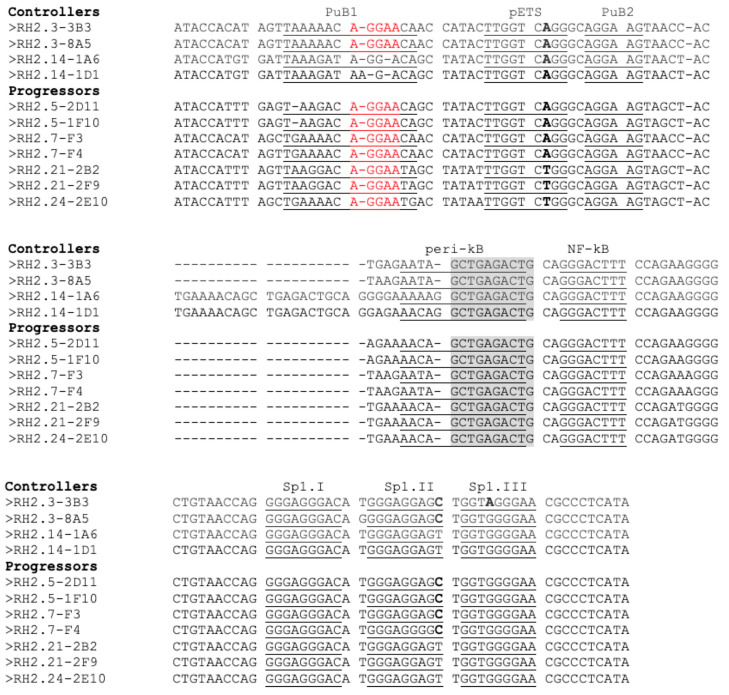
Alignment of the regulatory region of the HIV-2 LTR from controllers and progressors. Binding sites are indicated above the sequences. The AGGAA motif in PuB1 is marked in red. Sequence variations in the pETS and Sp1 motifs are shown in bold. The conserved 3′-half of the peri-kB site is highlighted in grey.

Given the high genetic diversity in the LTR U3 subregion, which encompasses most known transcription factor (TF) binding sites, we sought to investigate the function of the LTRs from controllers vs. progressors (Figure 5). We observed that the peri-ETS, PuB2, the 3′-half of the peri-kB, and the NF-kB sites were conserved. The essential motif AGGAA of the PuB site was conserved in PuB2 but was missing in sequences derived from participant #14 in PuB1. Despite this difference, Elf-1 was predicted to bind alternative motifs that did not contain the AGGAA but rather ATAAGA or AGGAC (https://jaspar.genereg.net, accessed on 25 October 2023). Thus, the PuB1 site was apparently functionally conserved in all sequences. Most disparities were found in a 21-nucleotide insertion between the PuB2 and peri-kB sites in viruses from donor #14. Similar insertions at the same location have been observed in Group B HIV-2 [57]. The three Sp1 binding sites were also conserved. The peri-ETS binding site is known to play a role in HIV-2 transcriptional regulation within activated CD4+ T-lymphocytes [58]. Importantly, variations in the LTR sequences did not consistently correlate with phenotypic differences in vivo. 

**Figure 6:**
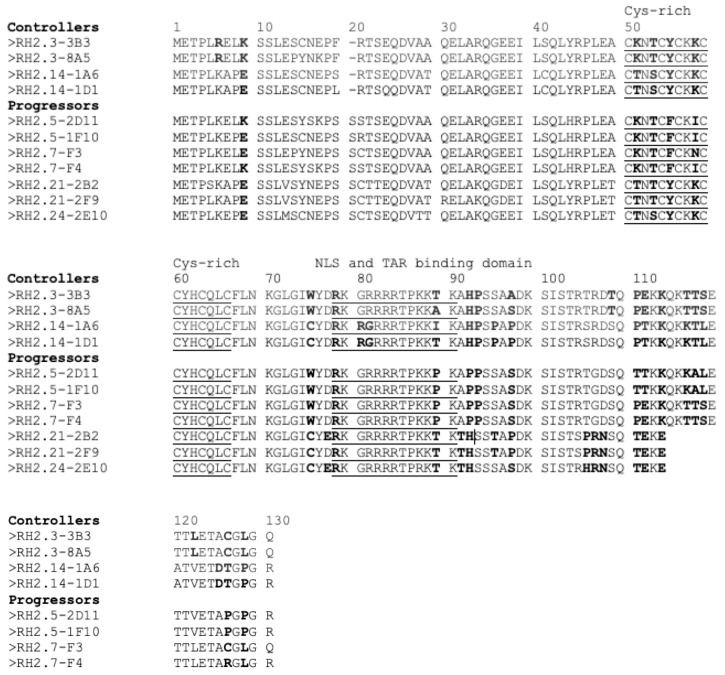
Alignment of HIV-2 Tat protein sequences from controllers and progressors. Cysteine-rich and the nuclear localization signal (NLS) and TAR-binding domains are underlined. Naturally polymorphic residues are in bold.

Reciprocally, an examination of Tat protein sequences showed that the seven cysteine residues crucial for Tat function were conserved across all viruses, and the core and basic regions, including the overlapping nuclear localization signal, and TAR-binding motifs were highly conserved (Figure 6).

**Figure 7:**
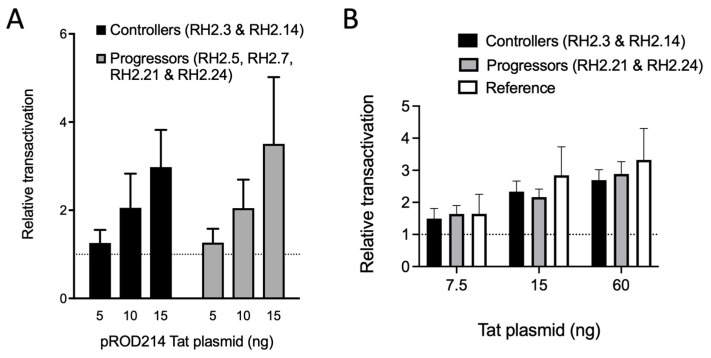
Conservation of transcriptional transactivation between HIV-2 controllers and progressors. (**A**) Relative transactivation of LTRs from controllers and progressors in response to co-transfection of 293T cells with various amounts of a plasmid encoding the Tat protein from the reference virus ROD. Basal transcription levels were arbitrarily set to a value of 1 (dashed line) and did not differ between controllers and progressors (not shown). (**B**) Relative transcriptional activity (in relative luminescence units, RLUs) of LTRs from controllers and progressors in response to co-transfection with various amounts of matched Tat from the same donor. Results obtained with LTR and Tat from the ROD reference virus (GenBank Accession ID:M15390) served as a control (“Reference”). Basal transcription levels were arbitrarily set to a value of 1 (dashed line) and did not differ between controllers and progressors.

However, given that transcriptional restriction appears to be important for HIV-2 control [33], we performed functional assays with LTRs of viruses isolated from the two controllers and two progressors, specifically RH2.21 and RH2.24 who yielded the fast replicating viruses. This selection aimed to increase the likelihood of detecting significant transcriptional differences. First, we assessed the basal transcriptional activity and Tat-mediated transactivation of cloned LTRs in cell-based assays and observed similar basal and Tat-enhanced transcriptional activity when using a plasmid expressing Tat from the reference strain (pROD214) (Figure 7A). 

Nonetheless, we considered that Tat transactivation potential may have co-evolved with the LTRs, thereby motivating the efforts to clone autologous Tats to compare LTR transactivation between controllers and progressors (Figure 7B). This work confirmed the absence of transcriptional differences between controllers and progressors in cell-based assays. 

## 4. Discussion

Our work possesses strengths and limitations. The viruses we employed exhibited substantial similarity. Cohort homogeneity serves as an advantage, as participants hailed from the same epidemiological region and had group A viruses, potentially facilitating the detection of genotypic differences linked to HIV-2 control. Conversely, this uniformity might have hindered our ability to observe phenotypic disparities in transcriptional regulation. Additionally, our participant count is limited due to the original cohort’s small size, compounded by the unavailability of older samples from the 2000s for analysis. The close kinship of viruses from controllers and progressors herein cautions against extrapolating specific amino acid roles to a particular biological phenotype.

Given that our previous study of the replicative capacity of viruses isolated from controllers or progressors showed discrepancies in replication that occurred through ex vivo infections of CD8-depleted PBMCs, such differences must arise autonomously from a retroviral factor and cell-intrinsic mechanisms. Specifically, HIV-2 isolated from controllers displayed lower replicative capacity than those derived from progressors [32]. To pinpoint the cause for lower replication in viruses from controllers, this study comprehensively characterized near full-length genome sequences of HIV-2. Our sequencing results comprise genes that were previously studied and independently confirmed previous findings regarding Vpx and Nef [37,38]. In addition, we found no genetic aberrations in Env, Nef, Rev, or Tat (Figure 2, Figure 3 and Figure 6). The conservation of Env agrees with our previous work that showed the functionality of receptor and coreceptor binding and entry [36]. 

HIV-2 viruses isolated from controllers display lower replicative capacity than those derived from progressors despite similar quantities of integrated DNA between controllers and progressors [27,29]. Thus, we postulated that this disparity might be linked to transcriptional deficiencies and examined the basal and Tat-enhanced transcriptional activity of LTRs from controllers and progressors (Figure 7). We carried this out both with a generic Tat and with matched Tat proteins from the same LTR donor. While prior studies have explored HIV-2 LTR transcriptional activity within group A and between groups A and B [57,59,60,61], a comparison between controllers and progressors was not undertaken before. Our study showed that the LTRs from controllers and progressors were similarly transcriptionally active and responsive to Tat (Figure 7) in the 293T transfection system that we used. We acknowledge that the Tat-LTR interactions and transcription regulation may be different in other cell types, such as CD4+ T-lymphocytes or monocytes. For example, it has been shown that the peri-kB sites have regulatory functions in monocytes but not T-lymphocytes [58]. Thus, our results may have been different in other cell types. However, the phenotypic results obtained with 293T cells agree with the genetic conservation of transcription factor binding sites in the LTR and functional domains within Tat of both groups of viruses used in this study. 

Given the consistent integrated DNA quantities between matched individuals, and the observed similarities in transcriptional activity between HIV-2 from controllers and progressors, we hypothesize that quality of integrant rather than their quantities may differ between controllers and progressors. It has been established that the nature of integration sites is pivotal for proviral transcription and replication-competent retrovirus production [4,62]. Hence, based on our findings, it is conceivable that dissimilar integration sites among controllers and progressors may contribute to their distinct phenotypes. 

**Figure 8:**
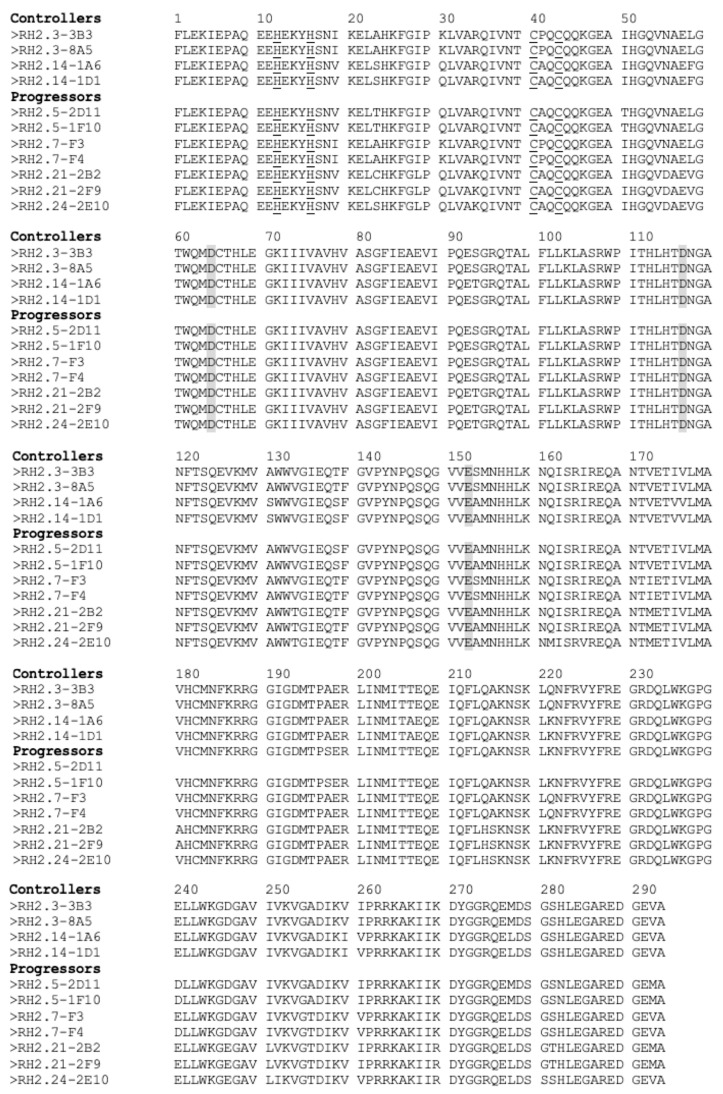
Alignment of HIV-2 integrase protein sequences from controllers and progressors. The zinc-binding domain is underlined. The D-D-E catalytic triad is shaded.

**Figure 9:**
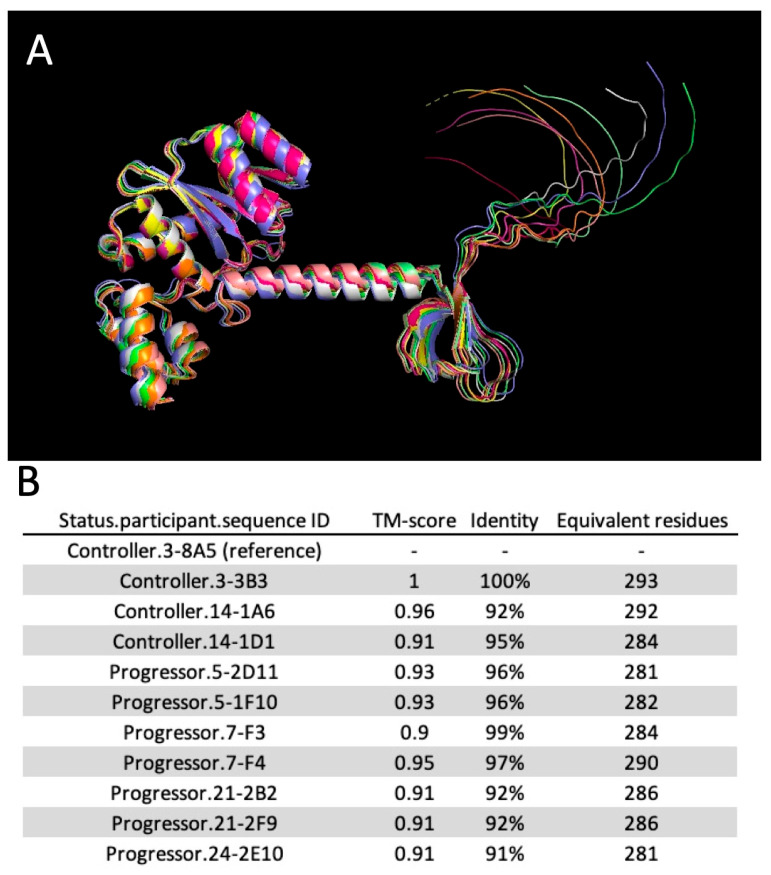
Comparison of integrase structures. (**A**) Pairwise alignment of all integrase in silico structures generated from the controllers and progressors of this study. Only the unstructured C-termini showed significant structural deviation (right side). (**B**) Pairwise scoring of integrase structure alignments. Sequence 8A5 from controller RH2.3 was used as a reference. TM-score, identity, and equivalent residues were quantified.

Integrase, the pivotal retroviral enzyme in the integration process, logically emerges as a potential influencer of integration disparities that, in turn, determine HIV-2 control or progression. Despite detecting several natural polymorphisms in integrase sequences, we did not pinpoint specific changes in integrase amino acid composition correlating with control or progression (Figure 8). The gross structure of integrase was also unchanged between progressors and controllers (Figure 9). Hence, functional differences might be mediated by combined polymorphisms within integrase itself or across various retroviral proteins. One attractive candidate for such co-regulation is Tat, given recent evidence that Tat and integrase bind cooperatively with TAR in HIV-1 [63]. We anticipate that this process is conserved in HIV-2. Our future work will focus on the characterization of HIV-2 integration in controllers and progressors.

## 5. Conclusions

Overall, the near full genome sequences of different HIV-2 viruses did not reveal unique features that explain the differences in cell-based replication capacity or in vivo progressive pathogenesis. Nevertheless, our limited set does not permit to exclude that viral factors may still be important and further research is warranted. 

## Figures and Tables

**Table 1:** Overview of participants’ characteristics.

Status, Participant ID and Year	CD4+ T-Cell Count	Viral Load	Viral Isolates (Replication Rate) ^1^
**Controllers:**			
**RH2.3**			
2000	770	<50	3B3/3C3/8A5 (low)
2016	580	<50	
**RH2.14**			
2000	550	<50	1D1/1A6 (low)
2015	650	<50	
**Progressors:**			
**RH2.5**			
1997	120	110,000	1F10/2D11 (intermediate)
2016	370	<50 *	
**RH2.7**			
1996	10	>500	F3/F4 (intermediate)
2016	deceased	-	
**RH2.21**			
1998	60	59,000	2B2/2F9 (high)
2016	deceased	-	
**RH2.24**			
1999	70	23,000	2D8/2E10 (high)
2016	lost-to-follow-up	-	

^1^ Replication rates were determined in [33]. * Under antiretroviral therapy.

**Table 2:** Residues at capsid positions 119, 159, and 178 in controllers vs. progressors.

Status, Participant ID	119	159	178
**Controllers:**			
RH2.3	Proline	Proline	Proline
RH2.14	Alanine	Serine	Proline
**Progressors:**			
RH2.5	Alanine	Serine	Alanine
RH2.7	Alanine	Proline	Proline
RH2.21	Proline	Serine	Proline
RH2.24	Alanine	Serine	Proline

## Data Availability

All sequences were submitted to GenBank and are accessible (Accession numbers MF595854-MF595866). Virus stocks of these biological clones are available via the EVAg program (https://www.european-virus-archive.com, accessed on 8 November 2023) under Human Immunodeficiency Virus type II, RH-2-X with specific clone names as mentioned in Table 1.
